# Understanding of HLA-conferred susceptibility to chronic hepatitis B infection requires HLA genotyping-based association analysis

**DOI:** 10.1038/srep24767

**Published:** 2016-04-19

**Authors:** Nao Nishida, Jun Ohashi, Seik-Soon Khor, Masaya Sugiyama, Takayo Tsuchiura, Hiromi Sawai, Keisuke Hino, Masao Honda, Shuichi Kaneko, Hiroshi Yatsuhashi, Osamu Yokosuka, Kazuhiko Koike, Masayuki Kurosaki, Namiki Izumi, Masaaki Korenaga, Jong-Hon Kang, Eiji Tanaka, Akinobu Taketomi, Yuichiro Eguchi, Naoya Sakamoto, Kazuhide Yamamoto, Akihiro Tamori, Isao Sakaida, Shuhei Hige, Yoshito Itoh, Satoshi Mochida, Eiji Mita, Yasuhiro Takikawa, Tatsuya Ide, Yoichi Hiasa, Hiroto Kojima, Ken Yamamoto, Minoru Nakamura, Hiroh Saji, Takehiko Sasazuki, Tatsuya Kanto, Katsushi Tokunaga, Masashi Mizokami

**Affiliations:** 1 Department of Hepatic Disease, Research Center for Hepatitis and Immunology, National Center for Global Health and Medicine, Chiba 272-8516, Japan; 2 Department of Human Genetics, Graduate School of Medicine, The University of Tokyo, Tokyo 113-0033, Japan; 3 Department of Biological Sciences, Graduate School of Science, The University of Tokyo, Tokyo 113-0033, Japan; 4 Department of Hepatology and Pancreatology, Kawasaki Medical School, Okayama 701-0192, Japan; 5 Department of Gastroenterology, Kanazawa University Graduate School of Medicine, Ishikawa 920-0942, Japan; 6 Clinical Research Center, National Nagasaki Medical Center, Nagasaki 856-8562, Japan; 7 Department of Gastroenterology and Nephrology, Graduate School of Medicine, Chiba University, Chiba 263-0022, Japan; 8 Department of Gastroenterology, Graduate School of Medicine, The University of Tokyo, Tokyo 113-0033, Japan; 9 Division of Gastroenterology and Hepatology, Musashino Red Cross Hospital, Tokyo 180-0023, Japan; 10 Center for Gastroenterology, Teine Keijinkai Hospital, Hokkaido 006-0811, Japan; 11 Department of Medicine, Shinshu University School of Medicine, Nagano 390-0802, Japan; 12 Department of Gastroenterological Surgery I, Graduate School of Medicine, Hokkaido University, Hokkaido 060-0808, Japan; 13 Division of Hepatology, Saga Medical School, Saga 849-0937, Japan; 14 Department of Gastroenterology and Hepatology, Hokkaido University Graduate School of Medicine, Hokkaido 060-0808, Japan; 15 Department of Gastroenterology and Hepatology, Okayama University Graduate School of Medicine, Dentistry, and Pharmaceutical Sciences, Okayama 700-8558, Japan; 16 Department of Hepatology, Osaka City University Graduate School of Medicine, Osaka 558-8585, Japan; 17 Gastroenterology and Hepatology, Yamaguchi University Graduate School of Medicine, Yamaguchi 753-8511, Japan; 18 Department of Hepatology, Sapporo-Kosei General Hospital, Hokkaido 060-0033, Japan; 19 Molecular Gastroenterology and Hepatology, Kyoto Prefectural University of Medicine, Kyoto 602-0841, Japan; 20 Division of Gastroenterology and Hepatology, Saitama Medical University, Saitama 350-0495, Japan; 21 Department of Gastroenterology and Hepatology, National Hospital Organization Osaka National Hospital, Osaka 540-0006, Japan; 22 Division of Hepatology, Department of Internal Medicine, Iwate Medical University, Iwate 020-8505, Japan; 23 Division of Gastroenterology, Department of Medicine, Kurume University School of Medicine, Fukuoka 830-0011, Japan; 24 Department of Gastroenterology and Metabology, Ehime University Graduate School of Medicine, Ehime 791-0295, Japan; 25 HLA Foundation Laboratory, Kyoto 600-8813, Japan; 26 Department of Medical Biochemistry, Kurume University School of Medicine, Fukuoka 830-0011, Japan; 27 Institute for Advanced Study, Kyushu University, Fukuoka 819-0395, Japan

## Abstract

Associations of variants located in the *HLA* class II region with chronic hepatitis B (CHB) infection have been identified in Asian populations. Here, HLA imputation method was applied to determine *HLA* alleles using genome-wide SNP typing data of 1,975 Japanese individuals (1,033 HBV patients and 942 healthy controls). Together with data of an additional 1,481 Japanese healthy controls, association tests of six *HLA* loci including *HLA-A*, *C*, *B*, *DRB1*, *DQB1*, and *DPB1,* were performed. Although the strongest association was detected at a SNP located in the *HLA-DP* locus in a SNP-based GWAS using data from the 1,975 Japanese individuals, HLA genotyping-based analysis identified DQB1*06:01 as having the strongest association, showing a greater association with CHB susceptibility (OR = 1.76, *P* = 6.57 × 10^−18^) than any one of five *HLA-DPB1* alleles that were previously reported as CHB susceptibility alleles. Moreover, *HLA* haplotype analysis showed that, among the five previously reported *HLA-DPB1* susceptibility and protective alleles, the association of two *DPB1* alleles (DPB1*09:01, and *04:01) had come from linkage disequilibrium with *HLA-DR*-*DQ* haplotypes, DRB1*15:02-DQB1*06:01 and DRB1*13:02-DQB1*06:04, respectively. The present study showed an example that SNP-based GWAS does not necessarily detect the primary susceptibility locus in the *HLA* region.

Hepatitis B virus (HBV) is an infectious disease that has spread worldwide with an estimated 350 million chronically infected people. Some countries in Asia and Africa are known to be high endemicity areas where the prevalence of chronic hepatitis B (CHB) infection is over 8%. In Japan, chronic infection of an estimated 1.5 million people was caused by mother-to-child transmission, the reuse of syringes and needles, and sexually transmitted infections. Previous genome wide association studies (GWASs) have reported CHB susceptibility loci including *HLA-DP*, *HLA-DQ*, *EHMT2*, *TCF19*, *HLA-C*, *UBE2L3*, *CFB*, *NOTCH4*, *HLA-DOA*, and *CD40* in Asian populations^1^,^2^,^3^,^4^,^5^. Among CHB susceptibility loci, associations between polymorphisms within *HLA*-*DP* locus and CHB infection were replicated in Asian and Arabian populations, including Japanese, Han Chinese, Korean, Thai and Saudi Arabian populations^6^,^7^.

Previous reports revealed that polymorphisms within the *HLA-DP* and *HLA-DQ* loci were independently associated with CHB infection in the Japanese population^2^,^3^. *HLA* class II genes are known to be highly polymorphic, which means that there are many different subtypes (i.e. *HLA* alleles) in the different individuals inside a population. Therefore, *HLA* genotyping-based association analysis is necessary to comprehensively understand the associations between *HLA* genes and CHB infection. There have been no reports to clearly analyze the association of *HLA* genes with CHB infection. This is the first report to clearly show the associations of *HLA* class II genes with CHB infection using the emerging method of *HLA* imputation. The findings in this paper will be essential for future analysis to clarify the mechanisms of the immune recognition of HBV antigens by HLA class II molecules.

## Results and Discussions

The association of *HLA-DP* and *HLA-DQ* loci with CHB infection was replicated in a GWAS using 1,975 Japanese individuals (1,033 HBV patients and 942 healthy controls) (Supplementary Fig. 1). The top hit SNP rs2395309 is located 6.1 kb downstream of the *HLA-DPA1* gene (OR = 1.92; 95%CI = 1.68–2.20, P = 1.24 × 10^−21^). Moreover, an intron variant of the *HLA-DPB1* gene and a 24.0 kb upstream variant of the *HLA-DQB1* gene showed significant associations with CHB infection (rs9277496, OR = 1.78; 95%CI = 1.56–2.03, *P* = 6.17 × 10^−18^ for *HLA-DPB1*; rs9368737, OR = 1.63; 95%CI =  1.44–1.85, *P* = 3.17 × 10^−14^ for *HLA-DQB1*). However, none of the variants located in the non-*HLA* region, including the CHB susceptibility loci reported in previous GWASs, showed significant associations with CHB infection in the Japanese GWAS.

To investigate the relationship between *HLA-DP* variants (rs2395309 for *HLA-DPA1* and rs9277496 for *HLA-DPB1*) and the *HLA-DQB1* variant (rs9368737) and CHB susceptibility, we performed logistic regression analysis using the three associated SNPs as covariates. Significant associations of variants within the *HLA-DP* and *HLA-DQ* loci with CHB susceptibility were independently identified, as previously reported (Supplementary Table 1). In the regression analysis using three representative SNPs located in both *HLA-DP* and *HLA-DQ* regions as covariates, a number of SNPs located around the SNPs showed weakened (Supplementary Fig. 2). These results indicated that SNPs in *HLA-DP* and *HLA-DQ* regions were in strong linkage disequilibrium (LD) each other.

In order to clearly understand the associations of *HLA* genes with CHB infection, *HLA* genotyping has been considered as the next step, in which *HLA* alleles that will behave as functionally distinct HLA allotypes are determined. Here, instead of *HLA* genotyping, we performed statistical imputation of classical *HLA* alleles for six *HLA* loci including *HLA-A*, *C*, *B*, *DRB1*, *DQB1*, and *DPB1* using 1,975 genome wide SNP typing data as in our previous report^8^. The call rates and imputation accuracies for six *HLA* loci were evaluated in 417 Japanese healthy controls^9^, whose *HLA* genotypes were determined using a PCR sequence-specific oligonucleotide (PCR-SSO) method. When only samples with posterior probability of 0.5 or more were considered, the call rates and imputation accuracies had a range of 98.1–100% and 97.3–100%, respectively, across six *HLA* loci (Supplementary Table 2 and Supplementary Table 3). Higher accuracy was achieved compared to previous reports in Asian populations^10^,^11^. Although the HLA alleles were imputed with high accuracy in the present study, four *HLA* class I alleles were shown to have a discordant rate of over 0.5% (more than 5 discordant alleles out of a total of 417 *HLA* genotypes); HLA-A*24:20 (8 discordances), HLA-A*26:02 (5 discordances), HLA-C*03:04 (6 discordances), and HLA-C*08:03 (10 discordances). Therefore, these four alleles were excluded from the following association analyses to avoid false positives due to an error of imputation.

Tests of the association of *HLA* alleles for six *HLA* loci with CHB susceptibility was carried out using data from a total of 3,456 Japanese individuals consisting of 1,975 individuals whose *HLA* genotypes were estimated by HLA imputation, and 1,481 Japanese healthy individuals whose *HLA* genotypes were determined using the PCR-SSO method. After removing the defect data to compare OR of each *HLA* allele, *HLA* allele frequencies between 805 HBV patients and 2,278 healthy controls were compared for the six *HLA* loci (Supplementary Table 4–9). Significant associations after correction of the significance level by the total number of observed alleles (P < 0.05/144) were observed for a total of twenty alleles. Interestingly, the strongest association was observed for HLA-DQB1*06:01, which showed a greater association with CHB susceptibility than any one of five *HLA-DPB1* alleles that were previously reported as CHB susceptibility alleles (OR = 1.76; 95%CI =  1.55–2.01, *P* = 6.57 × 10^−18^ for DQB1*06:01).

As is well known, strong LD between *DRB1* and *DQB1* alleles and less strong LD between *DPB1* and *DRB1*-*DQB1* alleles/haplotypes have been reported in many populations^12^,^13^,^14^. Strong LD (r-squared and D prime) between *HLA* class II alleles was also observed in the studied Japanese individuals (Supplementary Table 10 and Supplementary Table 11). Haplotype frequencies for six *HLA* loci, for three *HLA* class I loci and for three *HLA* class II loci were estimated using the PHASE software and were compared between HBV patients and healthy controls (Supplementary Table 12, Supplementary Table 13 and Table 1). Among the twenty-five haplotypes of *HLA-A-C-B-DRB1-DQB1-DPB1* whose frequencies were over 0.5% in either of two groups (i.e. HBV patients and healthy controls), the most frequent haplotype showed the strongest association with CHB susceptibility in the studied individuals (OR = 1.81; 95%CI = 1.47–2.22, *P* = 1.03 × 10^−8^ for HLA-A*24:02-C*12:02-B*52:01-DRB1*15:02-DQB1*06:01-DPB1*09:01). Because the estimated haplotypes of six *HLA* loci were highly varied, subdivided haplotypes with low frequency may lead to difficulty in detection of a true association. Haplotype analysis of *HLA* class I genes and *HLA* class II genes showed a total of twenty-three haplotypes and twenty-five haplotypes, respectively, whose frequencies were over 1.0% in either of the two groups. Among these haplotypes, the haplotype harboring DQB1*06:01 showed up with the highest frequency in the studied individuals, and had a significant association with CHB susceptibility (OR = 1.91; 95%CI =  1.61–2.28, *P* = 1.13 × 10^−13^ for HLA-DRB1*15:02-DQB1*06:01-DPB1*09:01).

In the current study, SNP based association tests showed that the significant association of variants located in the *HLA* class II region with CHB susceptibility was replicated in Japanese individuals. Although *HLA-DQ* and *DP* were shown to be independently associated with CHB susceptibility by applying regression analysis with associated variants as covariates, further analysis of HLA molecules is necessary to clarify the pathogenesis of HBV infection. To clearly understand the associations of *HLA* genes with CHB infection, *HLA* alleles were determined by the HLA imputation method using the genome-wide SNP typing data set. *HLA* class II alleles showed stronger associations with CHB susceptibility than *HLA* class I alleles. Interestingly, HLA-DQB1*06:01 showed the strongest association out of a total of twenty associated alleles, including any one of the previously reported *HLA-DPB1* alleles (i.e. DPB1*05:01 and *09:01 for susceptibility to CHB infection; DPB1*02:01, *04:01, and *04:02 for protection against CHB infection).

**Figure 1:**
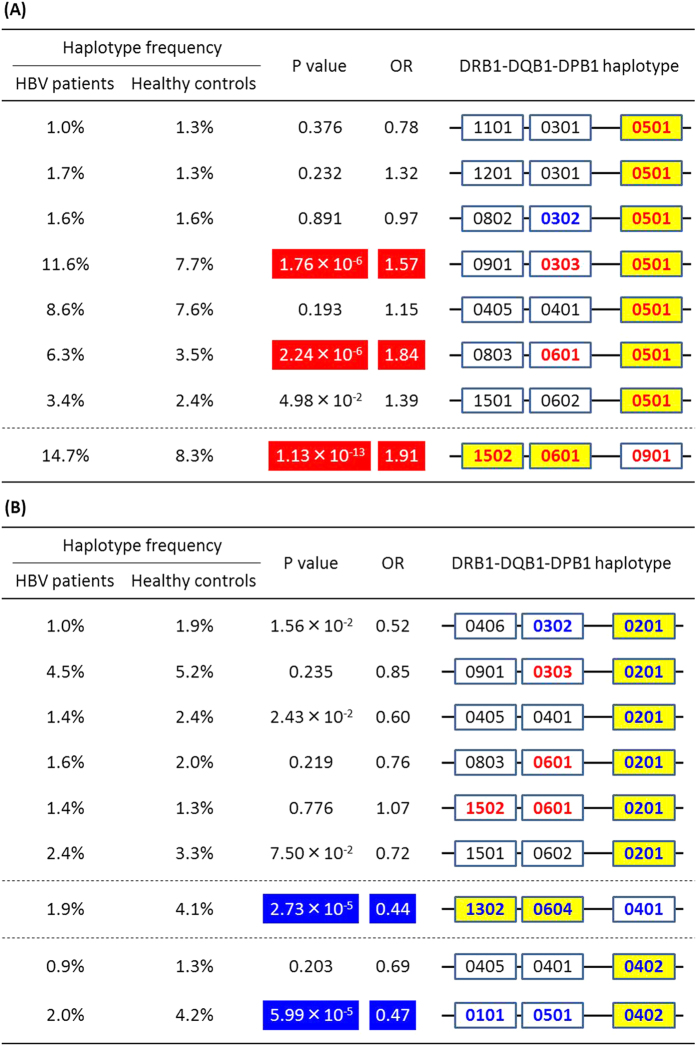
Associations of estimated haplotypes of *HLA* class II genes harboring. (**A**) *DPB1* alleles susceptible to chronic hepatitis B infection, and (**B**) *DPB1* alleles protective against chronic hepatitis B infection. Estimated haplotypes, whose frequencies were over 1% (**A**) in both of two groups, and (**B**) in either of two groups (i.e. HBV patients and healthy controls), are depicted with P values and OR. P values were calculated using Pearson’s chi-square test in the presence vs. the absence of each haplotype. *HLA* alleles that are significantly associated with CHB infection in single point analysis are depicted in bold red (susceptible) and bold blue (protective).

Haplotype analysis of *HLA* class II genes showed seven haplotypes that were significantly associated with susceptibility to or protection against CHB infection (Table 1). Figure 1A,B summarize the associations of each allele and estimated haplotypes of *HLA* class II genes with CHB susceptibility. A variety of haplotypes harboring DPB1*05:01 were observed. Of these, two haplotypes, DRB1*09:01-DQB1*03:03-DPB1*05:01 and DRB1*08:03-DQB1*06:01-DPB1*05:01, showed significant associations, with the same trend of association (i.e. susceptibility to CHB infection). These results imply that association of DPB1*05:01 may have the primary effect on CHB susceptibility, regardless of *DRB1* and *DQB1* alleles. The same can be said for haplotypes harboring DPB1*02:01 or *04:02, although no significant association with CHB infection was observed in haplotypes harboring DPB1*02:01.

Although haplotypes harboring DPB1*09:01 or DPB1*04:01 showed significant associations with susceptibility to or protection against CHB infection, respectively, the primary effect on CHB susceptibility may be explained by *DRB1*-*DQB1* haplotypes. As for the haplotype harboring DPB1*09:01, two haplotypes harboring the counterpart of DRB1*15:02-DQB1*06:01 were determined to have significant associations, with the same trend of association (i.e. susceptibility to CHB infection) (Table 1). The same can be said for the haplotype harboring DPB1*04:01. Two haplotypes harboring the counterpart of DRB1*13:02- DQB1*06:04 were determined to have signification associations, with the same trend of association (i.e. protection against CHB infection) (Table 1).

Associations of variants located in the *HLA* class II region with CHB susceptibility have been identified in several studies based on GWAS including the present study. Although *HLA-DR* and *DQ*, which are known to be in strong LD, and *HLA-DP* were independently associated with CHB susceptibility, it is difficult to clearly understand the association of *HLA* genes with CHB susceptibility using SNP based GWASs. Thus, the association of a specific SNP in the *HLA* region with CHB susceptibility may result from compositing effects of several *HLA* alleles. Therefore, the emerging method of HLA imputation, which uses a genome-wide SNP typing data set, is considered to be an effective strategy for comprehensive understanding of *HLA*-disease associations. Indeed, the present study showed that among the five previously reported *HLA-DPB1* susceptibility alleles, three *DPB1* alleles (DPB1*05:01, *02:01, and *04:02) had the primary effects on CHB susceptibility. However, the association of the remaining two alleles (DPB1*09:01 and *04:01) had come from LD with *HLA-DR*-*DQ* haplotypes (i.e. DRB1*15:02-DQB1*06:01 and DRB1*13:02-DQB1*06:04, respectively). These observations provide an example that SNP-based GWAS does not necessarily detect the primary susceptibility locus in this particular genomic region.

The disease-associated *HLA* alleles which were identified in this study may be beneficial to select patients who need a continuous follow-up (i.e. patients harboring susceptible *HLA* allele to CHB infection). As our current results showed, observed odds ratio of disease-associated *HLA* alleles were 1.91 for susceptible *DRB1-DQB1-DPB1* haplotype, and 0.44 for protective *DRB1-DQB1-DPB1* haplotype. Although the impact of disease-associated *HLA* alleles or haplotypes on clinical diagnosis is indeed small, further analysis to identify new host factors behind *HLA* genes, viral factors and clinical features may proceed effectively by selecting individuals who have the disease-associated *HLA* class II alleles.

## Methods

### Ethics approval

This study was approved by the Ethics Committee of The University of Tokyo and of all of the following Institutes and Hospitals throughout Japan that participated in this collaborative study: National Center for Global Health and Medicine, Kawasaki Medical School, Kanazawa University Graduate School of Medicine, National Nagasaki Medical Center, Chiba University, Musashino Red Cross Hospital, Nagoya City University Graduate School of Medical Sciences, Teine Keijinkai Hospital, Shinshu University School of Medicine, Hokkaido University, Saga Medical School, Hokkaido University Graduate School of Medicine, Okayama University Graduate School of Medicine, Osaka City University Graduate School of Medicine, Yamaguchi University Graduate School of Medicine, Kyoto Prefectural University of Medicine, Tottori University, Saitama Medical University, National Hospital Organization Osaka National Hospital, Iwate Medical University, Kurume University School of Medicine, Ehime University Graduate School of Medicine, Hyogo College of Medicine, and Kitasato University School of Medicine. All participants provided written informed consent for participation in this study and the methods were carried out in accordance with the approved guidelines.

### Genomic DNA samples and clinical data

Of the 3,456 Japanese genomic DNA samples used in this study, 1,975 samples were obtained from healthy volunteers (n = 942) or HBV patients (n = 1,033) at 28 multi-center hospitals (liver units with hepatologists) and universities throughout Japan; the other 1,481 samples were used in previous studies^15^,^16^. HBV status was determined based on serological results for hepatitis B surface antigen (HBsAg) and hepatitis B core antibody (anti-HBc) using a fully automated chemiluminescent enzyme immunoassay system (Abbott ARCHITECT; Abbott Japan, Tokyo, Japan, or LUMIPULSE f or G1200; Fujirebio, Inc., Tokyo, Japan). The unrelated and anonymized Japanese healthy control samples were collected from volunteers with/without HBV vaccination.

### SNP genotyping and data cleaning

For the GWAS, we genotyped 1,975 samples (1,033 Japanese HBV patients and 942 Japanese healthy controls) using the Affymetrix Axiom Genome-Wide ASI 1 Array, according to the manufacturer’s instructions. All samples had an overall call rate of more than 96%; the average overall call rate for HBV patients and healthy controls was 99.45% (97.48–99.84) and 99.31% (96.18–99.89), respectively. We then applied the following thresholds for SNP quality control during the data cleaning: SNP call rate ≥95%, minor allele frequency ≥5% in both HBV patients and healthy controls, and Hardy-Weinberg Equilibrium *P*-value ≥0.001 in healthy controls^17^. Of the SNPs on autosomal chromosomes, 424,157 SNPs passed the quality control filters and were used for the association analysis. All cluster plots for SNPs with a *P* < 0.0001 based on a chi-square test of the allele frequency model were checked by visual inspection, and SNPs with ambiguous genotype calls were excluded. Supplementary Fig. 1 shows the regional Manhattan plot of the HLA region (Chr6: 32,256,456 – 33,258,648, GRCh37 hg19).

### HLA imputation

SNP data from 1,975 samples were extracted from an extended MHC (xMHC) region ranging from 25759242 to 33534827 bp based on the hg19 position. We conducted 2-field HLA genotype imputation for six class I and class II *HLA* genes using the HIBAG R package^8^,^18^. For *HLA-A*, *B*, *DRB1*, *DQB1* and *DPB1*, our in-house Japanese imputation reference^8^ was used for HLA genotype imputation; for *HLA-C*, the HIBAG Asian reference^18^ was used for HLA genotype imputation. We applied post-imputation quality control using call-threshold (CT > 0.5); the call rate of the successfully imputed samples ranged from 98.1–100% for the 6 *HLA* classes we imputed. Quality of HLA imputation was further accessed using the data of 417 healthy controls in which their HLA genotypes were determined using the PCR-SSO method. In total, we imputed 148 *HLA* genotypes of *HLA* class I and class II genes.

### Haplotype estimation

The phased haplotypes consisting of six *HLA* loci were estimated by using the PHASE program version 2.1^19^,^20^. The estimated 6-locus haplotypes were further used for the estimation of haplotypes of three *HLA* class II loci (i.e., the collapsing method was applied to the phased data for six *HLA* loci).

### Pairwise LD between *HLA* class II alleles

The pairwise LD parameters, *r*^2^ and *D*′^21^, between alleles at different class II *HLA* loci were calculated based on the haplotype frequencies estimated by using the expectation maximization (EM) algorithm^22^. Here, each *HLA* allele was assumed to be one of the alleles at a bi-allelic locus, and the other *HLA* alleles at the same locus were assumed to be the other allele. For example, the DRB1*01:01 allele and the other *DRB1* alleles were designated as “A allele” and “B allele”, respectively. Accordingly, the EM algorithm for the estimation of haplotype frequencies for two loci each with two alleles could be applied to two *HLA* alleles at different loci.

### Association test

To assess the association of *HLA* allele or haplotype with CHB infection, Pearson’s chi-square test was applied to a two-by-two contingency table based on the allele or haplotype frequencies. The susceptibility to or resistance against CHB infection was evaluated based on the OR (i.e., OR >1 and OR < 1 indicate susceptible and resistant alleles, respectively). To avoid false positives due to multiple testing for 144 *HLA* alleles, the significance level was set at 0.00035 (=0.05/144).

## Additional Information

**How to cite this article**: Nishida, N. *et al.* Understanding of HLA-conferred susceptibility to chronic hepatitis B infection requires HLA genotyping-based association analysis. *Sci. Rep.* **6**, 24767; doi: 10.1038/srep24767 (2016).

## Supplementary Material

Supplementary Information

## Figures and Tables

**Table 1:** Haplotype analysis of *HLA* class II genes in HBV patients and healthy controls.

Haplotype	HBV patients	Healthy Controls	P-value*	OR		
	(2n = 1,610)	(2n = 4,556)			95% CI	
(*DRB1*-*DQB1*-*DPB1*)	%	%			Lower	Upper
**01:01-05:01-04:02**	**2**.**0**	**4**.**2**	**5**.**99E-05**	**0**.**47**	**0**.**33**	**0**.**69**
04:03-03:02-05:01	0.5	1.1	2.74E-02	0.44	0.21	0.93
04:05-04:01-02:01	1.4	2.4	2.43E-02	0.60	0.38	0.94
04:05-04:01-03:01	1.2	0.7	4.17E-02	1.78	1.01	3.12
04:05-04:01-04:02	0.9	1.3	2.03E-01	0.69	0.39	1.22
04:05-04:01-05:01	8.6	7.6	1.93E-01	1.15	0.93	1.41
04:06-03:02-02:01	1.0	1.9	1.56E-02	0.52	0.31	0.89
08:02-03:02-05:01	1.6	1.6	8.91E-01	0.97	0.61	1.53
08:03-06:01-02:01	1.6	2.0	2.19E-01	0.76	0.48	1.18
08:03-06:01-02:02	2.2	1.7	1.42E-01	1.35	0.90	2.01
**08:03-06:01-05:01**	**6**.**3**	**3**.**5**	**2**.**24E-06**	**1**.**84**	**1**.**42**	**2**.**38**
09:01-03:03-02:01	4.5	5.2	2.35E-01	0.85	0.65	1.11
**09:01-03:03-05:01**	**11**.**6**	**7**.**7**	**1**.**76E-06**	**1**.**57**	**1**.**31**	**1**.**90**
11:01-03:01-05:01	1.0	1.3	3.76E-01	0.78	0.45	1.36
12:01-03:01-05:01	1.7	1.3	2.32E-01	1.32	0.83	2.10
12:01-03:03-05:01	1.1	0.7	1.15E-01	1.61	0.89	2.93
12:02-03:01-05:01	1.6	0.9	2.21E-02	1.76	1.08	2.89
**13:02-06:04-04:01**	**1**.**9**	**4**.**1**	**2**.**73E-05**	**0**.**44**	**0**.**30**	**0**.**66**
**13:02-06:04-05:01**	**0**.**1**	**1**.**1**	**3**.**52E-04**	**0**.**12**	**0**.**03**	**0**.**48**
14:05-05:03-05:01	1.5	0.9	6.89E-02	1.59	0.96	2.63
15:01-06:02-02:01	2.4	3.3	7.50E-02	0.72	0.51	1.03
15:01-06:02-05:01	3.4	2.4	4.98E-02	1.39	1.00	1.93
15:02-06:01-02:01	1.4	1.3	7.76E-01	1.07	0.66	1.76
**15:02-06:01-05:01**	**1**.**9**	**0**.**9**	**4**.**98E-04**	**2**.**27**	**1**.**41**	**3**.**66**
**15:02-06:01-09:01**	**14**.**7**	**8**.**3**	**1**.**13E-13**	**1**.**91**	**1**.**61**	**2**.**28**

The estimated haplotype frequencies over 1.0% in either of two groups (i.e. HBV patients and healthy controls) are shown in the table.

^*^P value was calculated by Pearson’s chi-square test in presence vs. absence of each haplotype. P values and OR, statistically significant after correction of the significance level (P < 0.05/25), are indicated in bold.
