# Supplementary material for: Understanding of HLA-conferred susceptibility to chronic hepatitis B infection requires HLA genotyping-based association analysis
Source: Sci Rep. 2016 Apr 19;6:24767. doi: 10.1038/srep24767 (PMC4835786; doi:10.1038/srep24767)
Supplement: Supplementary Information [file srep24767-s1.doc]

**SUPPLEMENTARY MATERIAL**

Understanding of HLA-conferred susceptibility to chronic hepatitis B infection requires HLA genotyping-based association analysis

Nao Nishida^1,2*^, Jun Ohashi^3^, Seik-Soon Khor^2^, Masaya Sugiyama^1^, Takayo Tsuchiura^1^, Hiromi Sawai^2^, Keisuke Hino^4^, Masao Honda^5^, Shuichi Kaneko^5^, Hiroshi Yatsuhashi^6^, Osamu Yokosuka^7^, Kazuhiko Koike^8^, Masayuki Kurosaki^9^, Namiki Izumi^9^, Masaaki Korenaga^1^, Jong-Hon Kang^10^, Eiji Tanaka^11^, Akinobu Taketomi^12^, Yuichiro Eguchi^13^, Naoya Sakamoto^14^, Kazuhide Yamamoto^15^, Akihiro Tamori^16^, Isao Sakaida^17^, Shuhei Hige^18^, Yoshito Itoh^19^, Satoshi Mochida^20^, Eiji Mita^21^, Yasuhiro Takikawa^22^, Tatsuya Ide^23^, Yoichi Hiasa^24^, Hiroto Kojima^25^, Ken Yamamoto^26^, Minoru Nakamura^6^, Hiroh Saji^25^, Takehiko Sasazuki^27^, Tatsuya Kanto^1^, Katsushi Tokunaga^2^, and Masashi Mizokami^1^

Supplementary Figure 1. Regional Manhattan plot of the HLA-region (Chr6: 32,256,456 - 33,258,648, GRCh37 hg19) in a GWAS using the data of 1,033 HBV patients and 942 healthy controls.


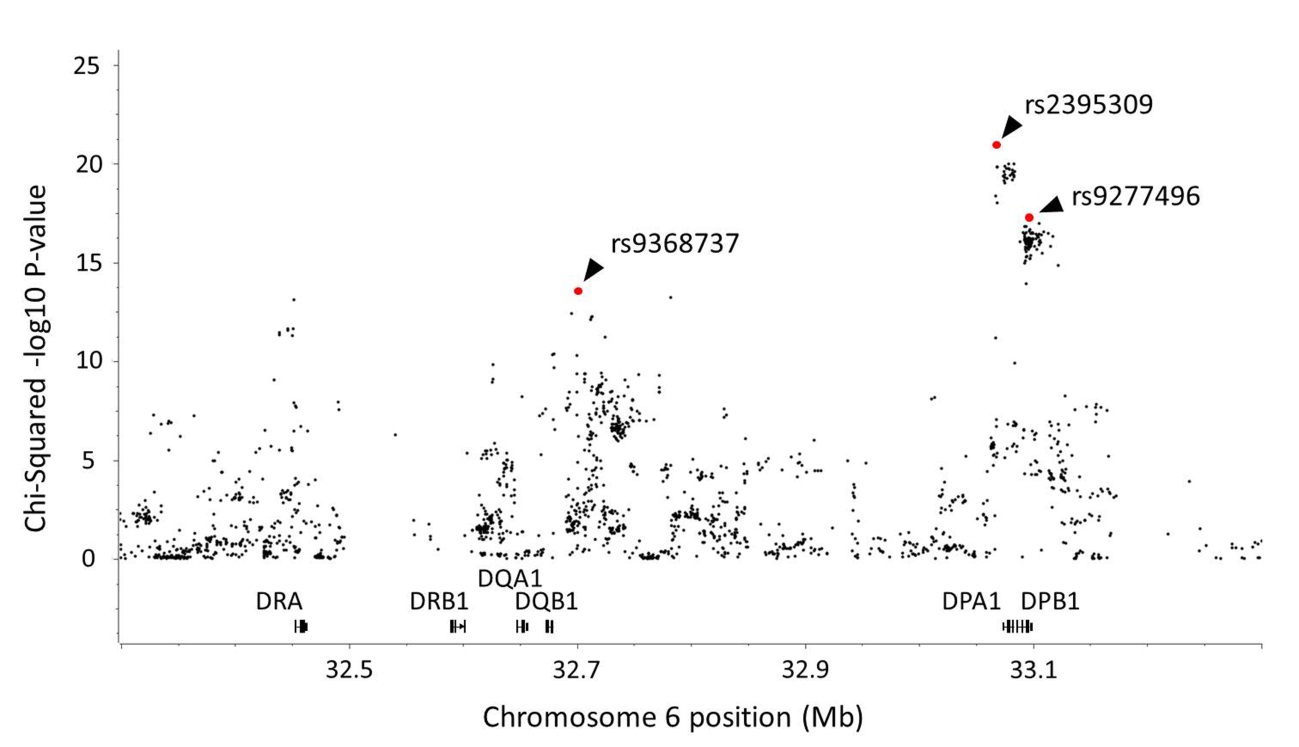


Supplementary Figure 2. Regional Manhattan plot of the HLA-region (Chr6: 32,256,456 - 33,258,648, GRCh37 hg19) applying a regression analysis with three SNPs (rs9368737, rs2395309, and 9277496) as covariates.


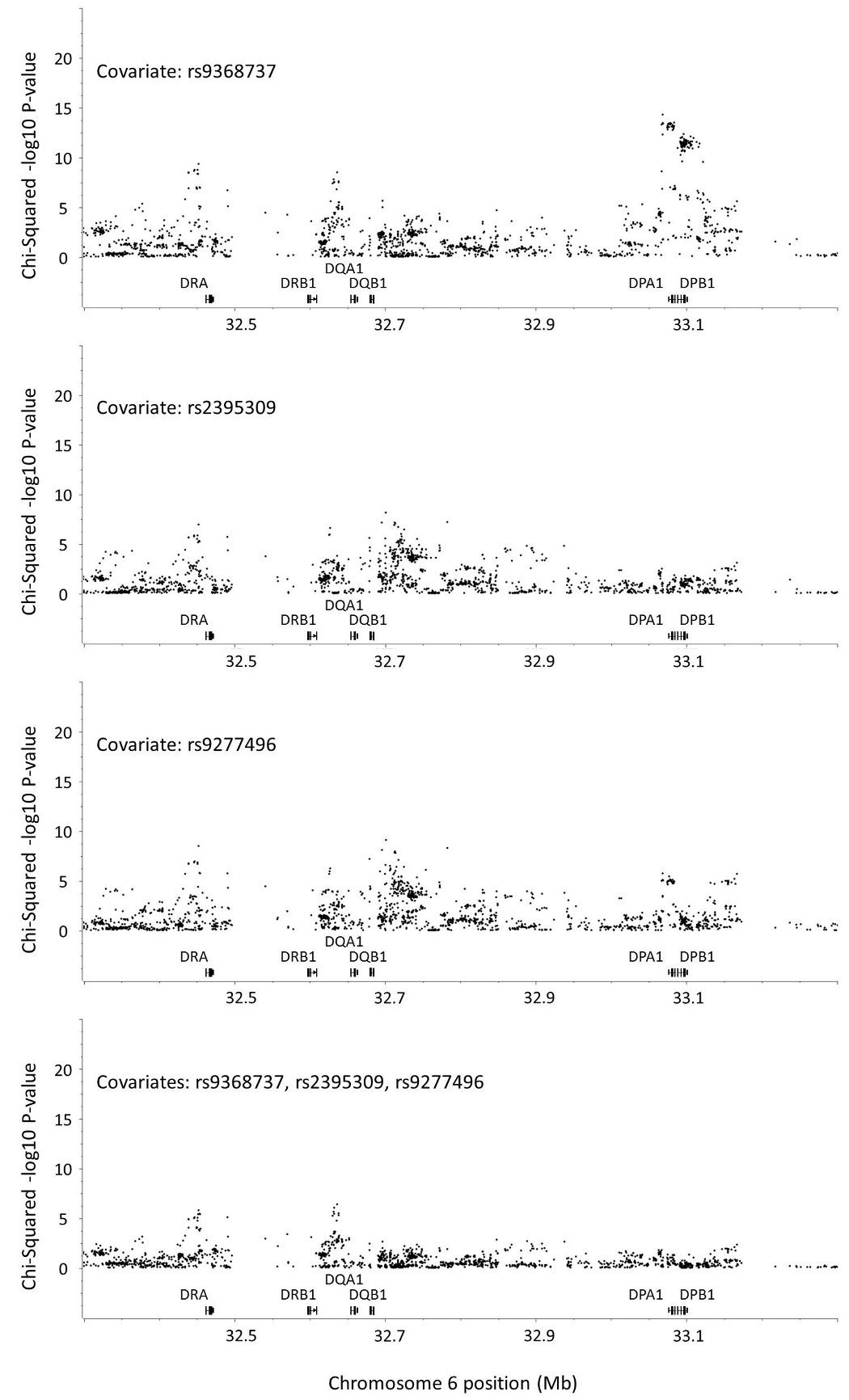


Supplementary Table 1. Logistic regression analysis of *HLA-DP* (rs2395309 and rs9277496) and *HLA-DQ* (rs9368737) with CHB susceptibility

| covariate | associated gene | *P_log_* | OR | 95%CI | |
| --- | --- | --- | --- | --- | --- |
|  |  |  |  | Lower | Upper |
| rs2395309 | *HLA-DPA1* | 1.31E-14 | 1.73 | 1.51 | 1.99 |
| rs9368737 | *HLA-DQB1* | 1.01E-08 | 1.48 | 1.30 | 1.70 |

| covariate | associated gene | *P_log_* | OR | 95%CI | |
| --- | --- | --- | --- | --- | --- |
|  |  |  |  | Lower | Upper |
| rs9277496 | *HLA-DPB1* | 8.97E-13 | 1.64 | 1.43 | 1.88 |
| rs9368737 | *HLA-DQB1* | 1.12E-09 | 1.52 | 1.33 | 1.74 |

Supplementary Table 2. HLA imputation for six *HLA* loci including *HLA-A*, *C*, *B*, *DRB1*, *DQB1* and *DPB1* in 417 Japanese healthy controls.

| Posterior probability* | *A* | *C* | *B* | *DRB1* | *DQB1* | *DPB1* |
| --- | --- | --- | --- | --- | --- | --- |
| 0 | 0 (0.0%) | 0 (0.0%) | 0 (0.0%) | 0 (0.0%) | 0 (0.0%) | 0 (0.0%) |
| 0-0.25 | 0 (0.0%) | 1 (0.2%) | 0 (0.0%) | 0 (0.8%) | 0 (0.0%) | 0 (0.0%) |
| 0.25-0.50 | 5 (1.2%) | 7 (1.7%) | 5 (1.2%) | 1 (0.2%) | 0 (0.0%) | 0 (0.0%) |
| 0.50-0.75 | 50 (12.0%) | 38 (9.1%) | 44 (10.6%) | 16 (3.8%) | 6 (1.4%) | 12 (2.9%) |
| 0.75-1 | 362 (86.8%) | 371 (89.0%) | 368 (88.2%) | 400 (95.9%) | 411 (98.6%) | 405 (97.1%) |

^*^The HIBAG R package recommends a posterior probability of 0.5 or more to remove poorly imputed HLA alleles.

Supplementary Table 3. Number of individuals with a discordant genotype in a comparison between HLA imputation and *HLA* genotyping in 417 Japanese healthy controls

| Posterior probability* (2n =834) | *A* | *C* | *B* | *DRB1* | *DQB1* | *DPB1* |
| --- | --- | --- | --- | --- | --- | --- |
| 0 | 0 / 0 | 0 / 0 | 0 / 0 | 0 / 0 | 0 / 0 | 0 / 0 |
| 0-0.25 | 0 / 0 | 1 / 2 | 0 / 0 | 0 / 0 | 0 / 0 | 0 / 0 |
| 0.25-0.50 | 3 / 10 | 1 / 14 | 3 / 10 | 1 / 2 | 0 / 0 | 0 / 0 |
| 0.50-0.75 | 14 / 100 | 9 / 76 | 4 / 88 | 0 / 32 | 0 / 12 | 0 / 24 |
| 0.75-1 | 7 / 724 | 13 / 742 | 5 / 736 | 0 / 800 | 0 / 822 | 0 / 810 |

^*^The HIBAG R package recommends a posterior probability of 0.5 or more to remove poorly imputed HLA alleles.

Supplementary Table 4. Associations of the *HLA-A* allele with CHB infection in Japanese individuals including 805 HBV patients and 2,278 healthy controls

| *HLA-A* |  | HBV patients | |  | Healthy controls | |  |  |  | OR |  |  |
| --- | --- | --- | --- | --- | --- | --- | --- | --- | --- | --- | --- | --- |
|  |  | (2n=1,610) | |  | (2n=4,556) | |  | P value^a^ |  |  | 95% CI | |
|  |  | count | % |  | count | % |  |  |  |  | Lower | Upper |
| 01:01 |  | 8 | 0.5 |  | 30 | 0.7 |  | 4.76E-01 |  | 0.75 | 0.34 | 1.65 |
| 02:01 |  | 173 | 10.7 |  | 463 | 10.2 |  | 5.09E-01 |  | 1.06 | 0.88 | 1.28 |
| 02:06 |  | 158 | 9.8 |  | 414 | 9.1 |  | 3.88E-01 |  | 1.09 | 0.90 | 1.32 |
| 02:07 |  | 65 | 4.0 |  | 156 | 3.4 |  | 2.55E-01 |  | 1.19 | 0.88 | 1.59 |
| 02:10 |  | 0 | 0.0 |  | 18 | 0.4 |  | 1.15E-02 |  | - | - | - |
| 02:18 |  | 0 | 0.0 |  | 2 | 0.0 |  | 4.00E-01 |  | - | - | - |
| 03:01 |  | 2 | 0.1 |  | 16 | 0.4 |  | 1.47E-01 |  | 0.35 | 0.08 | 1.54 |
| 03:02 |  | 0 | 0.0 |  | 4 | 0.1 |  | 2.34E-01 |  | - | - | - |
| 11:01 |  | 139 | 8.6 |  | 421 | 9.2 |  | 4.66E-01 |  | 0.93 | 0.76 | 1.13 |
| 11:02 |  | 0 | 0.0 |  | 5 | 0.1 |  | 1.84E-01 |  | - | - | - |
| 24:02 |  | 616 | 38.3 |  | 1714 | 37.6 |  | 6.49E-01 |  | 1.03 | 0.91 | 1.16 |
| 24:05 |  | 0 | 0.0 |  | 1 | 0.0 |  | 5.52E-01 |  | - | - | - |
| 24:07 |  | 0 | 0.0 |  | 1 | 0.0 |  | 5.52E-01 |  | - | - | - |
| 24:08 |  | 0 | 0.0 |  | 4 | 0.1 |  | 2.34E-01 |  | - | - | - |
| 24:20^b^ |  | 0 | 0.0 |  | 22 | 0.5 |  | NA |  | NA | NA | NA |
| 26:01 |  | 136 | 8.4 |  | 347 | 7.6 |  | 2.86E-01 |  | 1.12 | 0.91 | 1.38 |
| 26:02^b^ |  | 1 | 0.1 |  | 66 | 1.4 |  | NA |  | NA | NA | NA |
| 26:03 |  | 35 | 2.2 |  | 105 | 2.3 |  | 7.62E-01 |  | 0.94 | 0.64 | 1.39 |
| 26:05 |  | 0 | 0.0 |  | 4 | 0.1 |  | 2.34E-01 |  | - | - | - |
| 30:01 |  | 0 | 0.0 |  | 4 | 0.1 |  | 2.34E-01 |  | - | - | - |
| 31:01 |  | 160 | 9.9 |  | 379 | 8.3 |  | 4.80E-02 |  | 1.22 | 1.00 | 1.48 |
| 33:03 |  | 117 | 7.3 |  | 380 | 8.3 |  | 1.74E-01 |  | 0.86 | 0.69 | 1.07 |

The susceptibility to or resistance against CHB infection was evaluated based on the OR (i.e., OR > 1 and OR < 1 indicate susceptible and resistant alleles, respectively).

^a^P value was calculated by Pearson’s chi-square test in presence vs. absence of each genotype. P values and OR, statistically significant after correction of the significance level (P < 0.05/144), are indicated in bold.

^b^Association test was not performed since the discordant rate was over 0.5%

Supplementary Table 5. Associations of the *HLA-C* allele with CHB infection in Japanese individuals including 805 HBV patients and 2,278 healthy controls

| *HLA-C* |  | HBV patients | |  | Healthy controls | |  |  |  | OR |  |  |
| --- | --- | --- | --- | --- | --- | --- | --- | --- | --- | --- | --- | --- |
|  |  | (2n=1,610) | |  | (2n=4,556) | |  | P value^a^ |  |  | 95% CI | |
|  |  | count | % |  | count | % |  |  |  |  | Lower | Upper |
| 01:02 |  | 302 | 18.8 |  | 827 | 18.2 |  | 5.89E-01 |  | 1.04 | 0.90 | 1.20 |
| 01:03 |  | 0 | 0.0 |  | 16 | 0.4 |  | 1.73E-02 |  | - | - | - |
| 02:02 |  | 0 | 0.0 |  | 1 | 0.0 |  | 5.52E-01 |  | - | - | - |
| 03:02 |  | 9 | 0.6 |  | 31 | 0.7 |  | 6.02E-01 |  | 0.82 | 0.39 | 1.73 |
| 03:03 |  | 206 | 12.8 |  | 539 | 11.8 |  | 3.07E-01 |  | 1.09 | 0.92 | 1.30 |
| 03:04^b^ |  | 234 | 14.5 |  | 561 | 12.3 |  | NA |  | NA | NA | NA |
| 03:43 |  | 0 | 0.0 |  | 1 | 0.0 |  | 5.52E-01 |  | - | - | - |
| 04:01 |  | 50 | 3.1 |  | 209 | 4.6 |  | 1.08E-02 |  | 0.67 | 0.49 | 0.91 |
| 05:01 |  | 2 | 0.1 |  | 16 | 0.4 |  | 1.47E-01 |  | 0.35 | 0.08 | 1.54 |
| 06:02 |  | 7 | 0.4 |  | 31 | 0.7 |  | 2.79E-01 |  | 0.64 | 0.28 | 1.45 |
| 07:01 |  | 0 | 0.0 |  | 4 | 0.1 |  | 2.34E-01 |  | - | - | - |
| **07:02** |  | **149** | **9.3** |  | **576** | **12.6** |  | **2.86E-04** |  | **0.70** | **0.58** | **0.85** |
| 07:04 |  | 16 | 1.0 |  | 44 | 1.0 |  | 9.22E-01 |  | 1.03 | 0.58 | 1.83 |
| 07:15 |  | 0 | 0.0 |  | 1 | 0.0 |  | 5.52E-01 |  | - | - | - |
| 08:01 |  | 87 | 5.4 |  | 344 | 7.6 |  | 3.69E-03 |  | 0.70 | 0.55 | 0.89 |
| 08:03^b^ |  | 0 | 0.0 |  | 53 | 1.2 |  | NA |  | NA | NA | NA |
| **12:02** |  | **303** | **18.8** |  | **546** | **12.0** |  | **7.79E-12** |  | **1.70** | **1.46** | **1.99** |
| 12:03 |  | 0 | 0.0 |  | 5 | 0.1 |  | 1.84E-01 |  | - | - | - |
| 14:02 |  | 117 | 7.3 |  | 272 | 6.0 |  | 6.58E-02 |  | 1.23 | 0.99 | 1.55 |
| 14:03 |  | 77 | 4.8 |  | 336 | 7.4 |  | 3.48E-04 |  | 0.63 | 0.49 | 0.81 |
| 15:02 |  | 51 | 3.2 |  | 143 | 3.1 |  | 9.54E-01 |  | 1.01 | 0.73 | 1.40 |

The susceptibility to or resistance against CHB infection was evaluated based on the OR (i.e., OR > 1 and OR < 1 indicate susceptible and resistant alleles, respectively).

^a^P value was calculated by Pearson’s chi-square test in presence vs. absence of each genotype. P values and OR, statistically significant after correction of the significance level (P < 0.05/144), are indicated in bold.

^b^Association test was not performed since the discordant rate was over 0.5%

Supplementary Table 6. Associations of the *HLA-B* allele with CHB infection in Japanese individuals including 805 HBV patients and 2,278 healthy controls

| *HLA-B* |  | HBV patients | |  | Healthy controls | |  |  |  | OR |  |  |
| --- | --- | --- | --- | --- | --- | --- | --- | --- | --- | --- | --- | --- |
|  |  | (2n=1,610) | |  | (2n=4,556) | |  | P value^*^ |  |  | 95% CI | |
|  |  | count | % |  | count | % |  |  |  |  | Lower | Upper |
| **07:02** |  | **47** | **2.9** |  | **286** | **6.3** |  | **2.98E-07** |  | **0.45** | **0.33** | **0.61** |
| 07:15 |  | 0 | 0.0 |  | 1 | 0.0 |  | 5.52E-01 |  | - | - | - |
| 13:01 |  | 28 | 1.7 |  | 62 | 1.4 |  | 2.77E-01 |  | 1.28 | 0.82 | 2.01 |
| 13:02 |  | 0 | 0.0 |  | 6 | 0.1 |  | 1.45E-01 |  | - | - | - |
| 15:01 |  | 103 | 6.4 |  | 350 | 7.7 |  | 8.95E-02 |  | 0.82 | 0.65 | 1.03 |
| 15:02 |  | 0 | 0.0 |  | 2 | 0.0 |  | 4.00E-01 |  | - | - | - |
| 15:07 |  | 5 | 0.3 |  | 21 | 0.5 |  | 4.23E-01 |  | 0.67 | 0.25 | 1.79 |
| 15:11 |  | 8 | 0.5 |  | 32 | 0.7 |  | 3.77E-01 |  | 0.71 | 0.32 | 1.54 |
| 15:18 |  | 21 | 1.3 |  | 79 | 1.7 |  | 2.41E-01 |  | 0.75 | 0.46 | 1.22 |
| 15:27 |  | 0 | 0.0 |  | 2 | 0.0 |  | 4.00E-01 |  | - | - | - |
| 15:28 |  | 0 | 0.0 |  | 2 | 0.0 |  | 4.00E-01 |  | - | - | - |
| 27:04 |  | 0 | 0.0 |  | 6 | 0.1 |  | 1.45E-01 |  | - | - | - |
| 27:05 |  | 0 | 0.0 |  | 4 | 0.1 |  | 2.34E-01 |  | - | - | - |
| 35:01 |  | 122 | 7.6 |  | 344 | 7.6 |  | 9.72E-01 |  | 1.00 | 0.81 | 1.24 |
| 35:05 |  | 0 | 0.0 |  | 1 | 0.0 |  | 5.52E-01 |  | - | - | - |
| 37:01 |  | 7 | 0.4 |  | 24 | 0.5 |  | 6.54E-01 |  | 0.82 | 0.35 | 1.92 |
| 38:01 |  | 0 | 0.0 |  | 1 | 0.0 |  | 5.52E-01 |  | - | - | - |
| 38:02 |  | 0 | 0.0 |  | 6 | 0.1 |  | 1.45E-01 |  | - | - | - |
| 39:01 |  | 52 | 3.2 |  | 144 | 3.2 |  | 8.92E-01 |  | 1.02 | 0.74 | 1.41 |
| 39:02 |  | 0 | 0.0 |  | 7 | 0.2 |  | 1.16E-01 |  | - | - | - |
| 39:04 |  | 0 | 0.0 |  | 12 | 0.3 |  | 3.93E-02 |  | - | - | - |
| 39:23 |  | 0 | 0.0 |  | 1 | 0.0 |  | 5.52E-01 |  | - | - | - |
| 40:01 |  | 105 | 6.5 |  | 239 | 5.2 |  | 5.52E-02 |  | 1.26 | 0.99 | 1.60 |
| 40:02 |  | 145 | 9.0 |  | 321 | 7.0 |  | 1.05E-02 |  | 1.31 | 1.06 | 1.60 |
| 40:03 |  | 4 | 0.2 |  | 21 | 0.5 |  | 2.49E-01 |  | 0.54 | 0.18 | 1.57 |
| 40:06 |  | 53 | 3.3 |  | 235 | 5.2 |  | 2.29E-03 |  | 0.63 | 0.46 | 0.85 |
| 40:52 |  | 0 | 0.0 |  | 2 | 0.0 |  | 4.00E-01 |  | - | - | - |
| 44:02 |  | 2 | 0.1 |  | 16 | 0.4 |  | 1.47E-01 |  | 0.35 | 0.08 | 1.54 |
| **44:03** |  | **77** | **4.8** |  | **337** | **7.4** |  | **3.15E-04** |  | **0.63** | **0.49** | **0.81** |
| 45:01 |  | 0 | 0.0 |  | 1 | 0.0 |  | 5.52E-01 |  | - | - | - |
| 46:01 |  | 116 | 7.2 |  | 237 | 5.2 |  | 2.94E-03 |  | 1.41 | 1.12 | 1.78 |
| 46:02 |  | 0 | 0.0 |  | 1 | 0.0 |  | 5.52E-01 |  | - | - | - |
| 48:01 |  | 31 | 1.9 |  | 131 | 2.9 |  | 4.05E-02 |  | 0.66 | 0.45 | 0.99 |
| 51:01 |  | 150 | 9.3 |  | 371 | 8.1 |  | 1.46E-01 |  | 1.16 | 0.95 | 1.41 |
| 51:02 |  | 3 | 0.2 |  | 12 | 0.3 |  | 5.90E-01 |  | 0.71 | 0.20 | 2.51 |
| **52:01** |  | **302** | **18.8** |  | **543** | **11.9** |  | **6.90E-12** |  | **1.71** | **1.46** | **1.99** |
| 52:11 |  | 0 | 0.0 |  | 1 | 0.0 |  | 5.52E-01 |  | - | - | - |
| 54:01 |  | 133 | 8.3 |  | 346 | 7.6 |  | 3.90E-01 |  | 1.10 | 0.89 | 1.35 |
| **55:02** |  | **9** | **0.6** |  | **108** | **2.4** |  | **4.66E-06** |  | **0.23** | **0.12** | **0.46** |
| 55:04 |  | 1 | 0.1 |  | 6 | 0.1 |  | 4.76E-01 |  | 0.47 | 0.06 | 3.92 |
| 56:01 |  | 11 | 0.7 |  | 38 | 0.8 |  | 5.58E-01 |  | 0.82 | 0.42 | 1.60 |
| 56:03 |  | 8 | 0.5 |  | 11 | 0.2 |  | 1.12E-01 |  | 2.06 | 0.83 | 5.14 |
| 58:01 |  | 9 | 0.6 |  | 32 | 0.7 |  | 5.43E-01 |  | 0.79 | 0.38 | 1.67 |
| 59:01 |  | 32 | 2.0 |  | 101 | 2.2 |  | 5.86E-01 |  | 0.89 | 0.60 | 1.34 |
| 67:01 |  | 26 | 1.6 |  | 53 | 1.2 |  | 1.66E-01 |  | 1.39 | 0.87 | 2.24 |

The susceptibility to or resistance against CHB infection was evaluated based on the OR (i.e., OR > 1 and OR < 1 indicate susceptible and resistant alleles, respectively).

^*^P value was calculated by Pearson’s chi-square test in presence vs. absence of each genotype. P values and OR, statistically significant after correction of the significance level (P < 0.05/144), are indicated in bold.

Supplementary Table 7. Associations of the *HLA-DRB1* allele with CHB infection in Japanese individuals including 805 HBV patients and 2,278 healthy controls

| *HLA-DRB1* |  | HBV patients | |  | Healthy controls | |  |  |  | OR |  |  |
| --- | --- | --- | --- | --- | --- | --- | --- | --- | --- | --- | --- | --- |
|  |  | (2n=1,610) | |  | (2n=4,556) | |  | P value^*^ |  |  | 95% CI | |
|  |  | count | % |  | count | % |  |  |  |  | Lower | Upper |
| **01:01** |  | **45** | **2.8** |  | **290** | **6.4** |  | **5.55E-08** |  | **0.42** | **0.31** | **0.58** |
| 01:02 |  | 0 | 0.0 |  | 1 | 0.0 |  | 5.52E-01 |  | - | - | - |
| 03:01 |  | 3 | 0.2 |  | 8 | 0.2 |  | 9.30E-01 |  | 1.06 | 0.28 | 4.01 |
| 04:01 |  | 22 | 1.4 |  | 47 | 1.0 |  | 2.72E-01 |  | 1.33 | 0.80 | 2.21 |
| 04:03 |  | 24 | 1.5 |  | 106 | 2.3 |  | 4.48E-02 |  | 0.64 | 0.41 | 0.99 |
| 04:04 |  | 0 | 0.0 |  | 7 | 0.2 |  | 1.16E-01 |  | - | - | - |
| 04:05 |  | 209 | 13.0 |  | 617 | 13.5 |  | 5.70E-01 |  | 0.95 | 0.80 | 1.13 |
| 04:06 |  | 29 | 1.8 |  | 148 | 3.2 |  | 2.80E-03 |  | 0.55 | 0.37 | 0.82 |
| 04:07 |  | 0 | 0.0 |  | 19 | 0.4 |  | 9.45E-03 |  | - | - | - |
| 04:10 |  | 26 | 1.6 |  | 87 | 1.9 |  | 4.49E-01 |  | 0.84 | 0.54 | 1.31 |
| 07:01 |  | 0 | 0.0 |  | 9 | 0.2 |  | 7.43E-02 |  | - | - | - |
| 08:02 |  | 56 | 3.5 |  | 188 | 4.1 |  | 2.51E-01 |  | 0.84 | 0.62 | 1.13 |
| 08:03 |  | 178 | 11.1 |  | 377 | 8.3 |  | 8.03E-04 |  | 1.38 | 1.14 | 1.66 |
| 09:01 |  | 300 | 18.6 |  | 696 | 15.3 |  | 1.65E-03 |  | 1.27 | 1.09 | 1.47 |
| 10:01 |  | 7 | 0.4 |  | 19 | 0.4 |  | 9.25E-01 |  | 1.04 | 0.44 | 2.49 |
| 11:01 |  | 29 | 1.8 |  | 124 | 2.7 |  | 4.13E-02 |  | 0.66 | 0.44 | 0.99 |
| 12:01 |  | 69 | 4.3 |  | 153 | 3.4 |  | 8.60E-02 |  | 1.29 | 0.96 | 1.72 |
| 12:02 |  | 38 | 2.4 |  | 85 | 1.9 |  | 2.22E-01 |  | 1.27 | 0.86 | 1.87 |
| 13:01 |  | 1 | 0.1 |  | 24 | 0.5 |  | 1.17E-02 |  | 0.12 | 0.02 | 0.87 |
| **13:02** |  | **39** | **2.4** |  | **312** | **6.8** |  | **4.45E-11** |  | **0.34** | **0.24** | **0.47** |
| 14:02 |  | 0 | 0.0 |  | 2 | 0.0 |  | 4.00E-01 |  | - | - | - |
| 14:03 |  | 9 | 0.6 |  | 60 | 1.3 |  | 1.29E-02 |  | 0.42 | 0.21 | 0.85 |
| 14:05 |  | 34 | 2.1 |  | 84 | 1.8 |  | 5.00E-01 |  | 1.15 | 0.77 | 1.72 |
| **14:06** |  | **2** | **0.1** |  | **61** | **1.3** |  | **3.10E-05** |  | **0.09** | **0.02** | **0.38** |
| 14:07 |  | 0 | 0.0 |  | 7 | 0.2 |  | 1.16E-01 |  | - | - | - |
| 14:54 |  | 58 | 3.6 |  | 139 | 3.1 |  | 2.79E-01 |  | 1.19 | 0.87 | 1.62 |
| 15:01 |  | 122 | 7.6 |  | 352 | 7.7 |  | 8.48E-01 |  | 0.98 | 0.79 | 1.21 |
| **15:02** |  | **301** | **18.7** |  | **509** | **11.2** |  | **1.57E-14** |  | **1.83** | **1.56** | **2.14** |
| 16:02 |  | 9 | 0.6 |  | 25 | 0.5 |  | 9.62E-01 |  | 1.02 | 0.47 | 2.19 |

The susceptibility to or resistance against CHB infection was evaluated based on the OR (i.e., OR > 1 and OR < 1 indicate susceptible and resistant alleles, respectively).

^*^P value was calculated by Pearson’s chi-square test in presence vs. absence of each genotype. P values and OR, statistically significant after correction of the significance level (P < 0.05/144), are indicated in bold.

Supplementary Table 8. Associations of the *HLA-DQB1* allele with CHB infection in Japanese individuals including 805 HBV patients and 2,278 healthy controls

| *HLA-DQB1* |  | HBV patients | |  | Healthy controls | |  |  |  | OR |  |  |
| --- | --- | --- | --- | --- | --- | --- | --- | --- | --- | --- | --- | --- |
|  |  | (2n=1,610) | |  | (2n=4,556) | |  | P value^*^ |  |  | 95% CI | |
|  |  | count | % |  | count | % |  |  |  |  | Lower | Upper |
| 02:01 |  | 3 | 0.2 |  | 17 | 0.4 |  | 2.57E-01 |  | 0.50 | 0.15 | 1.70 |
| 03:01 |  | 153 | 9.5 |  | 502 | 11.0 |  | 8.98E-02 |  | 0.85 | 0.70 | 1.03 |
| **03:02** |  | **85** | **5.3** |  | **394** | **8.6** |  | **1.42E-05** |  | **0.59** | **0.46** | **0.75** |
| **03:03** |  | **325** | **20.2** |  | **737** | **16.2** |  | **2.49E-04** |  | **1.31** | **1.13** | **1.52** |
| 04:01 |  | 213 | 13.2 |  | 608 | 13.3 |  | 9.07E-01 |  | 0.99 | 0.84 | 1.17 |
| 04:02 |  | 47 | 2.9 |  | 179 | 3.9 |  | 6.39E-02 |  | 0.74 | 0.53 | 1.02 |
| **05:01** |  | **52** | **3.2** |  | **310** | **6.8** |  | **1.57E-07** |  | **0.46** | **0.34** | **0.62** |
| 05:02 |  | 38 | 2.4 |  | 97 | 2.1 |  | 5.86E-01 |  | 1.11 | 0.76 | 1.62 |
| 05:03 |  | 63 | 3.9 |  | 157 | 3.4 |  | 3.85E-01 |  | 1.14 | 0.85 | 1.54 |
| **06:01** |  | **479** | **29.8** |  | **883** | **19.4** |  | **6.57E-18** |  | **1.76** | **1.55** | **2.01** |
| 06:02 |  | 112 | 7.0 |  | 337 | 7.4 |  | 5.59E-01 |  | 0.94 | 0.75 | 1.17 |
| 06:03 |  | 1 | 0.1 |  | 23 | 0.5 |  | 1.42E-02 |  | 0.12 | 0.02 | 0.91 |
| **06:04** |  | **37** | **2.3** |  | **291** | **6.4** |  | **3.29E-10** |  | **0.34** | **0.24** | **0.49** |
| 06:09 |  | 2 | 0.1 |  | 21 | 0.5 |  | 5.68E-02 |  | 0.27 | 0.06 | 1.15 |

The susceptibility to or resistance against CHB infection was evaluated based on the OR (i.e., OR > 1 and OR < 1 indicate susceptible and resistant alleles, respectively).

^*^P value was calculated by Pearson’s chi-square test in presence vs. absence of each genotype. P values and OR, statistically significant after correction of the significance level (P < 0.05/144), are indicated in bold.

Supplementary Table 9. Associations of the *HLA-DPB1* allele with CHB infection in Japanese individuals including 805 HBV patients and 2,278 healthy controls

| *HLA-DPB1* |  | HBV patients | |  | Healthy controls | |  |  |  | OR |  |  |
| --- | --- | --- | --- | --- | --- | --- | --- | --- | --- | --- | --- | --- |
|  |  | (2n=1,610) | |  | (2n=4,556) | |  | P value^*^ |  |  | 95% CI | |
|  |  | count | % |  | count | % |  |  |  |  | Lower | Upper |
| **02:01** |  | **302** | **18.8** |  | **1111** | **24.4** |  | **3.87E-06** |  | **0.72** | **0.62** | **0.83** |
| 02:02 |  | 47 | 2.9 |  | 155 | 3.4 |  | 3.49E-01 |  | 0.85 | 0.61 | 1.19 |
| 03:01 |  | 84 | 5.2 |  | 197 | 4.3 |  | 1.40E-01 |  | 1.22 | 0.94 | 1.58 |
| **04:01** |  | **36** | **2.2** |  | **246** | **5.4** |  | **1.76E-07** |  | **0.40** | **0.28** | **0.57** |
| **04:02** |  | **90** | **5.6** |  | **425** | **9.3** |  | **3.16E-06** |  | **0.58** | **0.46** | **0.73** |
| **05:01** |  | **741** | **46.0** |  | **1756** | **38.5** |  | **1.46E-07** |  | **1.36** | **1.21** | **1.52** |
| 06:01 |  | 1 | 0.1 |  | 23 | 0.5 |  | 1.42E-02 |  | 0.12 | 0.02 | 0.91 |
| 08:01 |  | 0 | 0.0 |  | 1 | 0.0 |  | 5.52E-01 |  | - | - | - |
| **09:01** |  | **262** | **16.3** |  | **454** | **10.0** |  | **1.11E-11** |  | **1.76** | **1.49** | **2.07** |
| 13:01 |  | 27 | 1.7 |  | 81 | 1.8 |  | 7.91E-01 |  | 0.94 | 0.61 | 1.46 |
| 14:01 |  | 12 | 0.7 |  | 60 | 1.3 |  | 6.65E-02 |  | 0.56 | 0.30 | 1.05 |
| 17:01 |  | 0 | 0.0 |  | 3 | 0.1 |  | 3.03E-01 |  | - | - | - |
| 19:01 |  | 8 | 0.5 |  | 29 | 0.6 |  | 5.33E-01 |  | 0.78 | 0.36 | 1.71 |
| 36:01 |  | 0 | 0.0 |  | 3 | 0.1 |  | 3.03E-01 |  | - | - | - |
| 38:01 |  | 0 | 0.0 |  | 5 | 0.1 |  | 1.84E-01 |  | - | - | - |
| 41:01 |  | 0 | 0.0 |  | 6 | 0.1 |  | 1.45E-01 |  | - | - | - |
| 47:01 |  | 0 | 0.0 |  | 1 | 0.0 |  | 5.52E-01 |  | - | - | - |

The susceptibility to or resistance against CHB infection was evaluated based on the OR (i.e., OR > 1 and OR < 1 indicate susceptible and resistant alleles, respectively).

^*^P value was calculated by Pearson’s chi-square test in presence vs. absence of each genotype. P values and OR, statistically significant after correction of the significance level (P < 0.05/144), are indicated in bold.

Supplementary Table 10. Linkage disequilibrium between HLA class II alleles (r-squared) in HBV patients (**A**) and healthy controls (**B**).

| **A**. HBV patients | *DRB1* | | | | | | | | | | | *DQB1* | | | | | |
| --- | --- | --- | --- | --- | --- | --- | --- | --- | --- | --- | --- | --- | --- | --- | --- | --- | --- |
|  | **01:01* | **04:03* | **04:06* | **08:03* | **09:01* | **11:01* | **13:01* | **13:02* | **14:03* | **14:06* | **15:02* | **03:02* | **03:03* | **05:01* | **06:01* | **06:03* | **06:04* |
| *DQB1*03:02* | 0.00 | 0.27 | 0.33 | 0.01 | 0.01 | 0.00 | 0.00 | 0.00 | 0.00 | 0.00 | 0.00 |  | | | | | |
| *DQB1*03:03* | 0.01 | 0.00 | 0.00 | 0.03 | 0.90 | 0.00 | 0.00 | 0.00 | 0.00 | 0.00 | 0.06 |  |  |  |  |  |  |
| *DQB1*05:01* | 0.86 | 0.00 | 0.00 | 0.00 | 0.01 | 0.00 | 0.00 | 0.00 | 0.00 | 0.00 | 0.01 |  |  |  |  |  |  |
| *DQB1*06:01* | 0.01 | 0.00 | 0.00 | 0.29 | 0.10 | 0.01 | 0.00 | 0.01 | 0.00 | 0.00 | 0.54 |  |  |  |  |  |  |
| *DQB1*06:03* | 0.00 | 0.00 | 0.00 | 0.00 | 0.00 | 0.00 | 1.00 | 0.00 | 0.00 | 0.00 | 0.00 |  |  |  |  |  |  |
| *DQB1*06:04* | 0.00 | 0.00 | 0.00 | 0.00 | 0.00 | 0.00 | 0.00 | 0.95 | 0.00 | 0.00 | 0.01 |  |  |  |  |  |  |
| *DPB1*02:01* | 0.00 | 0.00 | 0.02 | 0.01 | 0.01 | 0.00 | 0.00 | 0.01 | 0.00 | 0.00 | 0.02 | 0.01 | 0.00 | 0.00 | 0.03 | 0.00 | 0.00 |
| *DPB1*04:01* | 0.00 | 0.00 | 0.00 | 0.00 | 0.00 | 0.00 | 0.00 | 0.63 | 0.00 | 0.00 | 0.01 | 0.00 | 0.00 | 0.00 | 0.01 | 0.00 | 0.67 |
| *DPB1*04:02* | 0.24 | 0.00 | 0.00 | 0.00 | 0.00 | 0.00 | 0.00 | 0.00 | 0.00 | 0.00 | 0.01 | 0.00 | 0.00 | 0.20 | 0.02 | 0.00 | 0.00 |
| *DPB1*05:01* | 0.01 | 0.00 | 0.00 | 0.00 | 0.03 | 0.00 | 0.00 | 0.02 | 0.01 | 0.00 | 0.12 | 0.00 | 0.04 | 0.01 | 0.05 | 0.00 | 0.02 |
| *DPB1*06:01* | 0.00 | 0.00 | 0.00 | 0.00 | 0.00 | 0.00 | 0.00 | 0.00 | 0.00 | 0.00 | 0.00 | 0.01 | 0.00 | 0.00 | 0.00 | 0.00 | 0.00 |
| *DPB1*09:01* | 0.01 | 0.00 | 0.00 | 0.02 | 0.04 | 0.00 | 0.00 | 0.00 | 0.00 | 0.00 | 0.69 | 0.00 | 0.04 | 0.01 | 0.38 | 0.00 | 0.00 |

| **B**. Healthy controls | *DRB1* | | | | | | | | | | | | *DQB1* | | | | | |
| --- | --- | --- | --- | --- | --- | --- | --- | --- | --- | --- | --- | --- | --- | --- | --- | --- | --- | --- |
|  | **01:01* | **04:03* | **04:06* | **04:07* | **08:03* | **09:01* | **11:01* | **13:01* | **13:02* | **14:03* | **14:06* | **15:02* | **03:02* | **03:03* | **05:01* | **06:01* | **06:03* | **06:04* |
| *DQB1*03:02* | 0.01 | 0.25 | 0.35 | 0.04 | 0.01 | 0.01 | 0.00 | 0.00 | 0.01 | 0.00 | 0.00 | 0.01 |  | | | | | |
| *DQB1*03:03* | 0.01 | 0.00 | 0.01 | 0.00 | 0.02 | 0.91 | 0.01 | 0.00 | 0.01 | 0.00 | 0.00 | 0.02 |  |  |  |  |  |  |
| *DQB1*05:01* | 0.93 | 0.00 | 0.00 | 0.00 | 0.01 | 0.01 | 0.00 | 0.00 | 0.00 | 0.00 | 0.00 | 0.01 |  |  |  |  |  |  |
| *DQB1*06:01* | 0.02 | 0.01 | 0.01 | 0.00 | 0.37 | 0.04 | 0.00 | 0.00 | 0.02 | 0.00 | 0.00 | 0.52 |  |  |  |  |  |  |
| *DQB1*06:03* | 0.00 | 0.00 | 0.00 | 0.00 | 0.00 | 0.00 | 0.00 | 0.96 | 0.00 | 0.00 | 0.00 | 0.00 |  |  |  |  |  |  |
| *DQB1*06:04* | 0.00 | 0.00 | 0.00 | 0.00 | 0.01 | 0.01 | 0.00 | 0.00 | 0.93 | 0.00 | 0.00 | 0.01 |  |  |  |  |  |  |
| *DPB1*02:01* | 0.01 | 0.00 | 0.02 | 0.00 | 0.00 | 0.01 | 0.00 | 0.00 | 0.01 | 0.00 | 0.00 | 0.01 | 0.01 | 0.01 | 0.01 | 0.01 | 0.00 | 0.01 |
| *DPB1*04:01* | 0.00 | 0.00 | 0.00 | 0.00 | 0.00 | 0.01 | 0.00 | 0.00 | 0.48 | 0.00 | 0.00 | 0.00 | 0.00 | 0.01 | 0.00 | 0.01 | 0.00 | 0.52 |
| *DPB1*04:02* | 0.30 | 0.00 | 0.00 | 0.00 | 0.01 | 0.00 | 0.00 | 0.00 | 0.00 | 0.00 | 0.00 | 0.01 | 0.01 | 0.01 | 0.29 | 0.01 | 0.00 | 0.00 |
| *DPB1*05:01* | 0.02 | 0.00 | 0.00 | 0.00 | 0.00 | 0.01 | 0.00 | 0.00 | 0.02 | 0.00 | 0.00 | 0.05 | 0.00 | 0.02 | 0.02 | 0.02 | 0.00 | 0.02 |
| *DPB1*06:01* | 0.00 | 0.00 | 0.00 | 0.00 | 0.00 | 0.00 | 0.00 | 0.00 | 0.00 | 0.00 | 0.00 | 0.00 | 0.00 | 0.00 | 0.00 | 0.00 | 0.00 | 0.00 |
| *DPB1*09:01* | 0.01 | 0.00 | 0.00 | 0.00 | 0.01 | 0.02 | 0.00 | 0.00 | 0.01 | 0.00 | 0.00 | 0.61 | 0.01 | 0.02 | 0.01 | 0.32 | 0.00 | 0.01 |

Supplementary Table 11. Linkage disequilibrium between HLA class II alleles (D prime) in HBV patients (**A**) and healthy controls (**B**).

| **A**. HBV patients | *DRB1* | | | | | | | | | | | *DQB1* | | | | | |
| --- | --- | --- | --- | --- | --- | --- | --- | --- | --- | --- | --- | --- | --- | --- | --- | --- | --- |
|  | **01:01* | **04:03* | **04:06* | **08:03* | **09:01* | **11:01* | **13:01* | **13:02* | **14:03* | **14:06* | **15:02* | **03:02* | **03:03* | **05:01* | **06:01* | **06:03* | **06:04* |
| *DQB1*03:02* | -1.00 | 1.00 | 1.00 | -1.00 | -1.00 | -0.04 | -1.00 | -1.00 | 0.01 | -1.00 | -0.62 |  | | | | | |
| *DQB1*03:03* | -1.00 | -1.00 | -1.00 | -1.00 | 1.00 | -1.00 | 1.00 | -0.67 | 0.07 | -1.00 | -1.00 |  |  |  |  |  |  |
| *DQB1*05:01* | 1.00 | -0.75 | -0.98 | -0.52 | -1.00 | -1.00 | -1.00 | 0.01 | -1.00 | -1.00 | -1.00 |  |  |  |  |  |  |
| *DQB1*06:01* | -1.00 | -0.84 | -0.70 | 1.00 | -1.00 | -1.00 | -1.00 | -1.00 | -1.00 | -0.56 | 1.00 |  |  |  |  |  |  |
| *DQB1*06:03* | -1.00 | -1.00 | -1.00 | -1.00 | -1.00 | -1.00 | 1.00 | -1.00 | -1.00 | -1.00 | -1.00 |  |  |  |  |  |  |
| *DQB1*06:04* | -0.07 | -0.19 | -1.00 | -1.00 | -0.75 | 0.06 | -1.00 | 1.00 | -1.00 | -1.00 | -1.00 |  |  |  |  |  |  |
| *DPB1*02:01* | -0.47 | 0.09 | 0.55 | -0.53 | 0.08 | 0.10 | -1.00 | -1.00 | 0.11 | -1.00 | -0.65 | 0.23 | 0.07 | 0.07 | -0.59 | -1.00 | -0.96 |
| *DPB1*04:01* | 0.03 | -0.14 | -1.00 | -0.88 | -0.69 | 0.03 | -1.00 | 0.83 | -1.00 | -1.00 | -1.00 | -1.00 | -0.49 | 0.02 | -0.82 | -1.00 | 0.83 |
| *DPB1*04:02* | 0.71 | 0.02 | -1.00 | -0.74 | -0.23 | 0.03 | -1.00 | -0.18 | -1.00 | -1.00 | -0.87 | -1.00 | -0.21 | 0.60 | -0.90 | -1.00 | -0.60 |
| *DPB1*05:01* | -0.55 | -0.59 | -0.51 | 0.17 | 0.35 | 0.18 | 1.00 | -1.00 | 1.00 | 1.00 | -0.78 | -0.05 | 0.37 | -0.59 | -0.38 | 1.00 | -1.00 |
| *DPB1*06:01* | -1.00 | -1.00 | -1.00 | -1.00 | -1.00 | -1.00 | -1.00 | -1.00 | -1.00 | -1.00 | -1.00 | 1.00 | -1.00 | -1.00 | -1.00 | -1.00 | -1.00 |
| *DPB1*09:01* | -1.00 | 0.11 | -0.12 | -1.00 | -0.93 | -1.00 | -1.00 | -1.00 | -0.80 | 0.26 | 0.90 | -0.31 | -0.88 | -1.00 | 0.90 | -1.00 | -1.00 |

| **B**. Healthy controls | *DRB1* | | | | | | | | | | | | *DQB1* | | | | | |
| --- | --- | --- | --- | --- | --- | --- | --- | --- | --- | --- | --- | --- | --- | --- | --- | --- | --- | --- |
|  | **01:01* | **04:03* | **04:06* | **04:07* | **08:03* | **09:01* | **11:01* | **13:01* | **13:02* | **14:03* | **14:06* | **15:02* | **03:02* | **03:03* | **05:01* | **06:01* | **06:03* | **06:04* |
| *DQB1*03:02* | -1.00 | 0.99 | 1.00 | 1.00 | -1.00 | -0.65 | -0.69 | -1.00 | -1.00 | -0.95 | -1.00 | -1.00 |  | | | | | |
| *DQB1*03:03* | -1.00 | -1.00 | -0.93 | -1.00 | -1.00 | 0.98 | -0.98 | -1.00 | -1.00 | -0.84 | -1.00 | -1.00 |  |  |  |  |  |  |
| *DQB1*05:01* | 1.00 | -1.00 | -1.00 | -1.00 | -1.00 | -1.00 | -1.00 | -1.00 | -0.86 | -1.00 | -1.00 | -1.00 |  |  |  |  |  |  |
| *DQB1*06:01* | -1.00 | -1.00 | -1.00 | -1.00 | 0.99 | -1.00 | -0.76 | -0.94 | -1.00 | -1.00 | -1.00 | 0.99 |  |  |  |  |  |  |
| *DQB1*06:03* | -1.00 | -1.00 | -0.69 | -1.00 | 0.01 | -1.00 | -1.00 | 1.00 | -0.79 | 0.06 | -1.00 | -1.00 |  |  |  |  |  |  |
| *DQB1*06:04* | -0.70 | -1.00 | -1.00 | -1.00 | -1.00 | -1.00 | -1.00 | -1.00 | 1.00 | -0.76 | -0.78 | -1.00 |  |  |  |  |  |  |
| *DPB1*02:01* | -0.61 | 0.13 | 0.46 | 0.53 | -0.13 | 0.13 | 0.08 | 0.35 | -0.62 | 0.12 | 0.03 | -0.49 | 0.21 | 0.11 | -0.46 | -0.33 | 0.39 | -0.62 |
| *DPB1*04:01* | -0.54 | -0.35 | -1.00 | 0.06 | -0.92 | -0.88 | -1.00 | -1.00 | 0.79 | -0.16 | -0.52 | -0.72 | -0.81 | -0.88 | -0.52 | -0.69 | -1.00 | 0.79 |
| *DPB1*04:02* | 0.68 | -1.00 | -1.00 | 0.16 | -0.79 | -0.51 | -1.00 | -0.55 | -0.50 | -0.85 | -0.57 | -0.79 | -0.89 | -0.54 | 0.63 | -0.78 | -0.53 | -0.46 |
| *DPB1*05:01* | -0.66 | 0.04 | -0.21 | -0.74 | 0.13 | 0.22 | 0.10 | 0.03 | -0.63 | 0.46 | 0.37 | -0.82 | 0.10 | 0.25 | -0.66 | -0.40 | 0.08 | -0.69 |
| *DPB1*06:01* | -0.68 | 0.09 | 0.04 | -1.00 | -1.00 | -0.36 | -1.00 | -1.00 | -0.79 | -1.00 | -1.00 | -0.51 | 0.13 | -0.82 | -0.78 | -0.87 | -1.00 | -0.69 |
| *DPB1*09:01* | -0.89 | -0.46 | -0.84 | -1.00 | -0.85 | -0.87 | -0.35 | 0.01 | -0.81 | -1.00 | -0.78 | 0.83 | -0.81 | -0.87 | -0.89 | 0.83 | -0.29 | -0.83 |

Supplementary Table 12. Haplotype analysis of *HLA-A-C-B-DRB1-DQB1-DPB1* in HBV patients and healthy controls.

| Haplotype (*A*-*C*-*B*-*DRB1*-*DQB1*-*DPB1*) |  | HBV patients |  | Healthy Controls |  |  |  |  |  |  |
| --- | --- | --- | --- | --- | --- | --- | --- | --- | --- | --- |
|  |  | (2n=1,610) |  | (2n=4,556) |  | P value* |  | OR | 95% CI | |
|  |  | % |  | % |  |  |  |  | Lower | Upper |
| 02:01-12:02-52:01-15:02-06:01-09:01 |  | 0.7 |  | 0.2 |  | 3.22E-03 |  | 3.48 | 1.44 | 8.40 |
| **02:06-01:02-54:01-04:05-04:01-05:01** |  | **0.9** |  | **0.1** |  | **5.80E-07** |  | **9.98** | **3.28** | **30.37** |
| 02:06-12:02-52:01-15:02-06:01-09:01 |  | 0.6 |  | 0.3 |  | 1.54E-01 |  | 1.82 | 0.79 | 4.22 |
| 02:07-01:02-46:01-08:03-06:01-02:02 |  | 0.7 |  | 0.9 |  | 6.73E-01 |  | 0.87 | 0.45 | 1.67 |
| 02:07-01:02-46:01-08:03-06:01-05:01 |  | 1.2 |  | 0.7 |  | 8.56E-02 |  | 1.64 | 0.93 | 2.89 |
| 11:01-01:02-54:01-04:05-04:01-05:01 |  | 0.5 |  | 0.8 |  | 1.77E-01 |  | 0.59 | 0.28 | 1.28 |
| 11:01-04:01-15:01-04:06-03:02-02:01 |  | 0.4 |  | 1.0 |  | 3.17E-02 |  | 0.43 | 0.19 | 0.95 |
| 24:02-01:02-46:01-08:03-06:01-02:02 |  | 0.7 |  | 0.5 |  | 2.86E-01 |  | 1.49 | 0.71 | 3.09 |
| 24:02-01:02-54:01-04:05-04:01-05:01 |  | 1.5 |  | 1.6 |  | 7.13E-01 |  | 0.92 | 0.58 | 1.46 |
| 24:02-01:02-59:01-04:05-04:01-04:02 |  | 0.2 |  | 0.6 |  | 6.66E-02 |  | 0.39 | 0.14 | 1.11 |
| **24:02-03:04-40:02-09:01-03:03-05:01** |  | **0.7** |  | **0.2** |  | **1.58E-03** |  | **3.91** | **1.57** | **9.74** |
| **24:02-07:02-07:02-01:01-05:01-04:02** |  | **1.1** |  | **2.6** |  | **6.32E-04** |  | **0.43** | **0.26** | **0.71** |
| 24:02-07:02-07:02-01:01-05:01-05:01 |  | 0.3 |  | 0.6 |  | 1.76E-01 |  | 0.52 | 0.20 | 1.36 |
| **24:02-08:01-40:06-09:01-03:03-05:01** |  | **0.9** |  | **0.2** |  | **6.51E-04** |  | **3.62** | **1.64** | **8.00** |
| 24:02-12:02-52:01-15:02-06:01-02:01 |  | 1.2 |  | 1.0 |  | 3.47E-01 |  | 1.29 | 0.76 | 2.20 |
| 24:02-12:02-52:01-15:02-06:01-05:01 |  | 1.5 |  | 0.7 |  | 5.75E-03 |  | 2.07 | 1.22 | 3.52 |
| **24:02-12:02-52:01-15:02-06:01-09:01** |  | **10.0** |  | **5.8** |  | **1.03E-08** |  | **1.81** | **1.47** | **2.22** |
| **24:02-14:02-51:01-09:01-03:03-05:01** |  | **0.7** |  | **0.2** |  | **2.31E-04** |  | **4.88** | **1.92** | **12.42** |
| 26:01-12:02-52:01-15:02-06:01-09:01 |  | 0.6 |  | 0.2 |  | 3.46E-02 |  | 2.56 | 1.04 | 6.30 |
| 31:01-12:02-52:01-15:02-06:01-09:01 |  | 0.6 |  | 0.5 |  | 4.35E-01 |  | 1.35 | 0.63 | 2.87 |
| 31:01-14:02-51:01-09:01-03:03-05:01 |  | 0.6 |  | 0.3 |  | 3.85E-02 |  | 2.37 | 1.02 | 5.49 |
| **33:03-14:03-44:03-08:03-06:01-05:01** |  | **1.1** |  | **0.3** |  | **1.92E-04** |  | **3.42** | **1.72** | **6.81** |
| 33:03-14:03-44:03-13:02-06:04-02:01 |  | 0.2 |  | 0.6 |  | 3.68E-02 |  | 0.30 | 0.09 | 0.99 |
| **33:03-14:03-44:03-13:02-06:04-04:01** |  | **1.6** |  | **3.3** |  | **5.85E-04** |  | **0.49** | **0.32** | **0.74** |
| 33:03-14:03-44:03-13:02-06:04-05:01 |  | 0.1 |  | 0.6 |  | 1.25E-02 |  | 0.19 | 0.05 | 0.81 |

The estimated haplotype frequencies over 0.5% in either of two groups (i.e. HBV patients and healthy controls) are shown in the table. The susceptibility to or resistance against CHB infection was evaluated based on the OR (i.e., OR > 1 and OR < 1 indicate susceptible and resistant haplotypes, respectively).

^*^P value was calculated by Pearson’s chi-square test in presence vs. absence of each haplotype. P values and OR, statistically significant after correction of the significance level (P < 0.05/25), are indicated in bold.

Supplementary Table 13. Haplotype analysis of *HLA-A-C-B* in HBV patients and healthy controls.

| Haplotype (*A*-*C*-*B*) |  | HBV patients |  | Healthy Controls |  |  |  |  |  |  |
| --- | --- | --- | --- | --- | --- | --- | --- | --- | --- | --- |
|  |  | (2n=1,610) |  | (2n=4,556) |  | P value* |  | OR | 95% CI | |
|  |  | % |  | % |  |  |  |  | Lower | Upper |
| 02:01-03:03-35:01 |  | 1.1 |  | 0.7 |  | 2.02E-01 |  | 1.46 | 0.81 | 2.63 |
| **02:06-01:02-54:01** |  | **1.3** |  | **0.3** |  | **1.01E-05** |  | **4.00** | **2.06** | **7.78** |
| 02:06-03:03-35:01 |  | 1.4 |  | 1.0 |  | 1.45E-01 |  | 1.45 | 0.88 | 2.41 |
| 02:06-07:02-39:01 |  | 1.2 |  | 1.0 |  | 3.47E-01 |  | 1.29 | 0.76 | 2.20 |
| 02:06-08:01-40:06 |  | 0.4 |  | 1.1 |  | 9.93E-03 |  | 0.34 | 0.15 | 0.80 |
| 02:07-01:02-46:01 |  | 3.5 |  | 2.5 |  | 2.55E-02 |  | 1.44 | 1.04 | 1.99 |
| 11:01-01:02-54:01 |  | 1.7 |  | 1.7 |  | 8.80E-01 |  | 0.97 | 0.62 | 1.50 |
| 11:01-04:01-15:01 |  | 1.4 |  | 2.0 |  | 1.75E-01 |  | 0.73 | 0.46 | 1.15 |
| 24:02-01:02-46:01 |  | 1.6 |  | 1.3 |  | 2.76E-01 |  | 1.30 | 0.81 | 2.07 |
| 24:02-01:02-54:01 |  | 3.0 |  | 3.4 |  | 4.40E-01 |  | 0.88 | 0.63 | 1.22 |
| 24:02-01:02-59:01 |  | 0.7 |  | 1.3 |  | 9.73E-02 |  | 0.59 | 0.32 | 1.11 |
| 24:02-03:03-35:01 |  | 1.2 |  | 1.3 |  | 9.24E-01 |  | 0.98 | 0.58 | 1.63 |
| 24:02-03:04-40:01 |  | 2.2 |  | 1.4 |  | 3.32E-02 |  | 1.56 | 1.03 | 2.34 |
| 24:02-03:04-40:02 |  | 1.7 |  | 1.6 |  | 8.02E-01 |  | 1.06 | 0.68 | 1.64 |
| **24:02-07:02-07:02** |  | **1.9** |  | **4.5** |  | **2.05E-06** |  | **0.40** | **0.27** | **0.59** |
| 24:02-08:01-40:06 |  | 1.1 |  | 1.1 |  | 8.90E-01 |  | 0.96 | 0.55 | 1.67 |
| **24:02-12:02-52:01** |  | **15.0** |  | **9.7** |  | **9.86E-09** |  | **1.63** | **1.38** | **1.93** |
| 24:02-14:02-51:01 |  | 2.4 |  | 1.7 |  | 9.98E-02 |  | 1.39 | 0.94 | 2.05 |
| 26:01-03:03-35:01 |  | 1.1 |  | 1.2 |  | 8.84E-01 |  | 0.96 | 0.56 | 1.64 |
| 26:01-03:04-40:02 |  | 2.2 |  | 1.5 |  | 6.34E-02 |  | 1.47 | 0.98 | 2.20 |
| 31:01-03:04-40:02 |  | 1.2 |  | 1.0 |  | 3.90E-01 |  | 1.26 | 0.74 | 2.14 |
| 31:01-14:02-51:01 |  | 2.5 |  | 2.0 |  | 2.01E-01 |  | 1.28 | 0.88 | 1.87 |
| 33:03-14:03-44:03 |  | 4.7 |  | 6.5 |  | 7.04E-03 |  | 0.70 | 0.54 | 0.91 |

The estimated haplotype frequencies over 0.5% in either of two groups (i.e. HBV patients and healthy controls) are shown in the table. The susceptibility to or resistance against CHB infection was evaluated based on the OR (i.e., OR > 1 and OR < 1 indicate susceptible and resistant haplotypes, respectively).

^*^P value was calculated by Pearson’s chi-square test in presence vs. absence of each haplotype. P values and OR, statistically significant after correction of the significance level (P < 0.05/23), are indicated in bold.
